# An exploration of the use of photobiomodulation for management of oral mucositis in children and young people undergoing cancer treatment in the UK

**DOI:** 10.1007/s00520-022-07450-3

**Published:** 2022-11-09

**Authors:** Claudia Heggie, Kara A. Gray-Burrows, Peter F. Day, Bob Phillips

**Affiliations:** 1 Paediatric Dentistry, University of Leeds, Leeds, UK; 2 Community Dental Service, Bradford District Care NHS Foundation Trust, Bradford, UK; 3 Paediatric Oncology, University of York, York, UK

**Keywords:** Photobiomodulation, Mucositis, Supportive care, Implementation, Paediatric

## Abstract

**Purpose:**

Oral mucositis affects up to 80% of children and young people (CYP) receiving chemotherapy. This can result in pain, reduced oral intake and, in severe cases, hospitalisation for parental nutrition and pain relief. Photobiomodulation is recommended by multiple bodies for mucositis management for those undergoing cancer treatments. The current use of photobiomodulation within the UK, and the barriers and facilitators to implementation is unknown.

**Method:**

An online mixed-methods survey was administered to representatives from the Children’s Cancer and Leukaemia Group (CCLG) between October 2021 and March 2022. This explored: use of photobiomodulation, planned future use, barriers and facilitators to implementation and dental assessment. Quantitative data underwent descriptive statistics. Barriers and facilitators to the implementation of photobiomodulation were analysed using the Theoretical Domains Framework (TDF).

**Results:**

All UK CCLG centres responded (*n* = 20, a response rate of 100%). Two units in Scotland were delivering photobiomodulation. A further four units were planning to implement a service. Most units, 65% (*n* = 13) utilised specialist Paediatric Dentistry services for dental assessment. In the TDF analysis, five domains were most frequently populated: knowledge, skills, environmental context and resources, social influences, and social/professional role and identity.

**Conclusion:**

Photobiomodulation was only available in Scotland in two children’s cancer units. Lack of knowledge and skills, and insufficient environmental resources were identified as barriers. Collaboration with paediatric dental services was identified as a facilitator. The establishment of a national network of Paediatric Dentists and Oncologists would promote collaboration to standardise protocols and to address the identified barriers to wider implementation of photobiomodulation.

## Introduction

Oral mucositis, the inflammation and/or ulceration of the oral mucosa, affects up to 80% of children and young people (CYP) receiving chemotherapy [1] and has a complex biopsychosocial impact on CYP and their parents [2]. In severe cases, CYP may require hospitalisation for parental nutrition and pain relief, which may delay scheduled chemotherapy [3]. Prevention and treatment of oral mucositis is therefore of high importance, both to patients and to oncology services to reduce impact on CYP, disruption to their cancer treatment, and subsequent increase in the cost and burden of cancer care [4].

Photobiomodulation (PBM) describes the delivery of low-level laser or non-coherent light source, such as light emitting diodes (LED), to alter cellular metabolism [5]. Red or near-infrared light is absorbed by the cells, specifically cytochrome c oxidase within the mitochondria [6]. This light absorption displaces nitric oxide from cytochrome c oxidase, resulting in increased ATP generation, activation of signalling pathways and transcription factors leading to increased expression of genes relating to cell proliferation, anti-inflammatory signally and anti-apoptotic proteins [6, 7]. The National Institute of Health and Care Excellence (NICE) and the Mucositis Study Group of the Multinational Association of Supportive Care in Cancer (MASCC)/International Society of Oral Oncology have published guidance recommending PBM for the prevention and treatment of oral mucositis following chemotherapy, radiotherapy and haematopoietic stem cell transplant [8–10]. However, almost all the evidence supporting these guidelines arises from adult populations. For CYP, the Pediatric Oncology Group of Ontario Mucositis Prevention Guideline Development Group recommend the use of this therapy for co-operative children receiving chemotherapy, haematopoietic stem cell transplant or head and neck radiotherapy [11, 12]. However, despite these recommendations, previous personal correspondence in 2018 found that within the UK, PBM was only available in Edinburgh and Glasgow for this population [13].

**Fig. 1:**
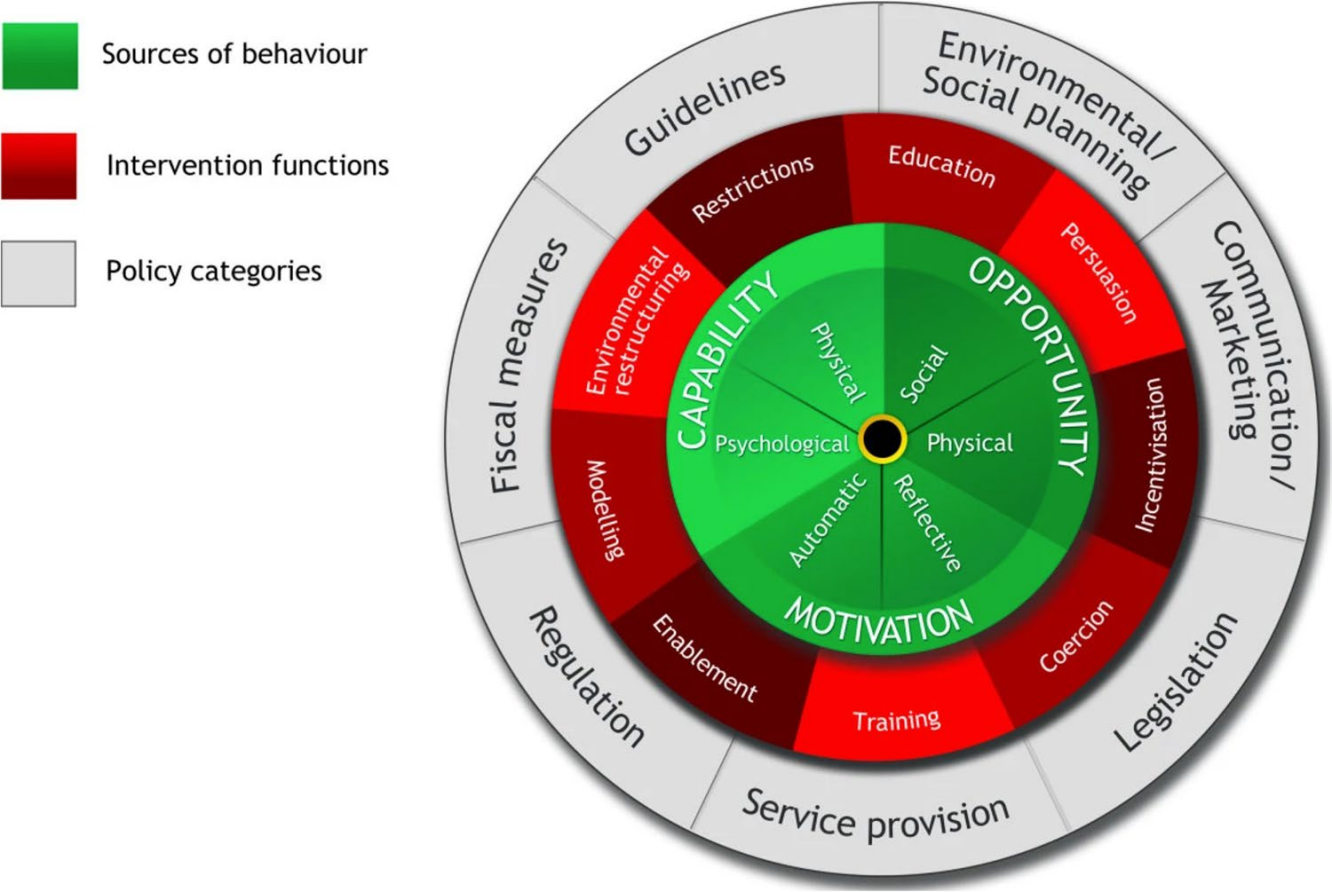
Behaviour Change Wheel reproduced from Michie, van Stralen and West (2011) [16]

Disparity often exists between evidence-based recommendations and healthcare delivered in practice [14]. Implementation research aims to promote uptake of evidence-based practice into routine care and explores the influences on healthcare professional and organisational behaviour [15]. The Behaviour Change Wheel was developed by Michie, van Stralen and West as a framework to characterise and design behaviour change interventions [16]. At the centre of the Behaviour Change Wheel lie sources of behaviour which can be categorised as Capability, Opportunity and Motivation behaviours within the COM-B system. Surrounding this hub, lie nine intervention functions which aim to change any deficits in the sources of behaviour. Situated on the outer ring of the Behaviour Change Wheel are seven policy categories that can enable intervention functions to occur (Fig. 1).

Implementation investigations can be broadly considered to consist of a “top-down” approach, for example, focusing on national policy, incentivisation systems and guidelines, or a “bottom-up” approach focusing on views of healthcare teams [17]. The Theoretical Domains Framework is a framework commonly utilised in such “bottom-up” approach implementation research [18]. This validated framework provides a structure to theoretically assess barriers and facilitators to implementation and consists of 14 domains: knowledge, skills, social/professional identity, beliefs about capabilities, optimism, beliefs about consequences, reinforcement, intentions, goals, memory attention and decision processes, environmental context and resources, social influences, emotion and behavioural regulation. The domains of the Theoretical Domains Framework align with the COM-B model, with each domain able to be categorised as associated with Capability, Opportunity or Motivation [19]. The Theoretical Domains Framework can be utilised to analyse these behaviours at an individual level as well as at a local organisational level. Additionally, it can be used to understand external factors influencing behaviour at an organisational or national policy level [19]. This provides an evidence informed approach to analysis of implementation barriers, therefore supporting development of interventions to overcome such barriers at the appropriate level.

## Aims

This cross-sectional survey aimed to expand on this initial scoping correspondence to provide data on provision of PBM, current practices and barriers and facilitators to implementation of this recommended therapy.

Additionally, to gain an understanding of the involvement of specialist Paediatric Dentistry services in dental assessment of these patients, as recommended in NHS commissioning standards in England [20].

## Method

An online questionnaire survey method was used following ethical approval from the University of Leeds Dental Research Ethics Committee (260,721/CH/331). The target population were representatives from the principal treatment centres in the UK within the Children’s Cancer and Leukaemia Group (CCLG) [21].

A blended questionnaire was piloted with Paediatric Dentists outside of the study population and finalised. The questionnaire explored: existing or planned future PBM delivery, patient selection, barriers and facilitators to implementation of a PBM service and dental assessment of Paediatric Oncology patients. Closed-ended questions were exhaustive and mutually exclusive. Open-ended questions were utilised to explore barriers and facilitators.

Participation was voluntary and participants were asked to identify the principal treatment centre where they worked. Participants were asked to respond to the survey only once. Survey administration occurred for a period of 5 months from October 2021 to March 2022. Where response could not be gained from Paediatric Oncology representatives, Paediatric Dentistry representatives with known links to the Oncology team were contacted given that PBM lies at the intersection of these specialties.

Data were extracted to Microsoft Excel®. Quantitative responses underwent descriptive statistical analysis. Free text responses relating to barriers and facilitators were analysed utilising the Theoretical Domains Framework by two researchers (CH & KG-B) with disagreements in coding resolved by discussion.

## Results

### Quantitative analysis

A response rate of 100% (*n* = 20) was achieved. One unit responded to the survey twice, and duplicate data were excluded from descriptive analysis.

Two units (10%) had an existing PBM service, and four further units (20%) had plans to implement this therapy (Table 1). Of those utilising PBM, CYP receiving PBM preventatively included those receiving chemotherapy prior to haematopoietic stem cell transplant, for osteosarcoma, non-Hodgkin’s lymphoma, and those with previous mucositis (with any cancer diagnosis). PBM was also used as a treatment in established mucositis in CYP from these groups.

**Table 1:** Distribution of existing and future planned PBM services within the Children’s Cancer and Leukaemia Group (CCLG)

Unit within Children’s Cancer and Leukaemia Group (CCLG)	Existing PBM service	Planned future implementation of PBM service
Addenbrooke’s Hospital, Cambridge	No	No
Alder Hey Children’s Hospital, Liverpool	No	No
Birmingham Children’s Hospital, Birmingham	No	No
Bristol Royal Hospital for Children, Bristol	No	No
Children’s Hospital, John Radcliffe Hospital, Oxford	No	No
Great North Children’s Hospital, Newcastle	No	Yes
Great Ormond Street Hospital for Children, London	No	No
Leeds Children’s Hospital, Leeds	No	Yes
Leicester Royal Infirmary, Leicester	No	Unsure
Noah’s Ark Children’s Hospital for Wales, Cardiff	No	No
Nottingham Children’s Hospital, Nottingham	No	Unsure
Royal Aberdeen Children’s Hospital, Aberdeen	No	No
Royal Belfast Hospital for Sick Children, Belfast	No	Unsure
Royal Hospital for Children, Glasgow	Yes	-
Royal Hospital for Sick Children, Edinburgh	Yes	-
Royal Manchester Children’s Hospital, Manchester	No	Yes
Royal Marsden Hospital, Sutton, Surrey	No	No
Sheffield Children’s Hospital, Sheffield	No	Yes
Southampton General Hospital, Southampton	No	Unsure
University College London Hospital, London	No	No

Staff involved with dental assessment of Paediatric Oncology patients were diverse and included: junior doctors (95% *n* = 19), nursing staff (95% *n* = 19), medical consultants (90% *n* = 18), advanced clinical practitioners (15% *n* = 3) and dentally qualified oral and maxillofacial surgery doctors (15% *n* = 3). From the 20 responders, two units (10%) involved general dental practitioners, and 65% (*n* = 13) involved specialist or consultant led Paediatric Dentistry services.

### Barriers and facilitators to the implementation

Most domains could be populated within the Theoretical Domains Framework, and data were attributed to levels of individual, local or national factors. Data were grouped into specific beliefs that provide detail regarding the influence of the domain on behaviour (Table 2). Descriptive overarching themes were then identified from the populated domains and collection of specific beliefs where data allowed.

**Table 2:** Data coded to Cane et al. (2012) Theoretical Domains Framework by domain and level. Grouped into specific beliefs and overarching themes where data allowed

Domain (definition)	Constructs	Overarching themes	Specific beliefs	Sample Quotes (level) Barriers	Quotes (level) Facilitators
Knowledge (An awareness of the existence of something)	Knowledge (including knowledge of condition/scientific rationale) Procedural knowledge Knowledge of task environment	Need for guidance to address lack of knowledge	I have limited knowledge of use of PBM for mucositis management	“Only heard about it as a possible thing – no plans to introduce and not sure how we’d go about it” *(local)* “Not aware of its use” *(local, national)* “Lack of awareness – unclear how would be implemented” *(individual, local, national)* “Aware of use in soft-tissue injury but not mucositis” *(individual)* “Lack of knowledge […]” *(individual, local, national)* “- Lack of awareness […]” (*individual, local, national)* “[…] Need SOP [Standard Operating Procedure] as not done before” *(local, national)*	
			I have limited knowledge of evidence-base in children	“Barriers: […] Lack of evidence base in children […]” *(individual)*	
			Guidance on use of PBM is needed	“[…] Lack of clear guidance” *(local, national)*	“Facilitators: […] evidence based practice & standardised protocols” (*local and national)*
			Knowledge is improved by having staff and teams with previous experience		“Improved identification and referral of patients to service” *(local)* “Facilitators- A member of staff with previous experience from another unit. […]” *(local)*
Skills (An ability or proficiency acquired through practice)	Skills Skills development Competence Ability Interpersonal skills Practice Skill assessment	Staff need access to training for skills acquisition	To implement a PBM service staff need to have access to training	“Barriers: sufficient numbers of trained staff” *(local)* “Barriers: […]access to relevant training” *(local, national)* “[…] – Infrastructure required (equipment, training, staff)” *(local, national)*	“Facilitators – enthusiastic technically competent staff and access to approved ‘kit’” *(local)*
			Skills development requires access to PBM equipment	“[…] – Infrastructure required (equipment, training, staff)” *(local, national)*	“Facilitators – enthusiastic technically competent staff and access to approved ‘kit’” *(local)*
			Standardised guidance and procedures would help skills development	“[…]Practicality – how and who. 3. Lack of clear guidance” *(local, national)* “[…] Need SOP [Standard Operating Procedure] as not done before” *(local, national)—skills development*	“Facilitators: […] standardised protocols” *(local, national)*
			Skilled staff with previous experience facilitate service implementation and development		“Facilitators- A member of staff with previous experience from another unit. […]” *(local)* “Improved identification and referral of patients to service” *(local)*
Social/professional role and identity (A coherent set of behaviours and displayed personal qualities of an individual in a social or work setting)	Professional identity Professional role Social identity Identity Professional boundaries Professional confidence Group identity Leadership Organisational commitment	Multiple, well identified groups need to work together towards implementation	Dental teams have a distinct role in implementation of a PBM service	“Staffing – […] prior was ad hoc” *(local)* “[…] Access to dental/appropriate team to administer […]” *(local)* “Would like to introduce but need to find dental team or others willing to be trained first! […]” *(local)*	“Staffing – more dental staff now available […]” *(local)* “Facilitators: very willing and keen dental colleagues, […]” *(local)* “Strong dental links, […]” *(local)* “Refer to Glasgow if required” *(local)*
			I am unsure who would deliver PBM	“not sure who would be responsible (nursing? medical?)” *(local)* “[…]Relevant professionals […]” *(local)* “[…] Access to dental/appropriate team to administer […]” *(local)* “Practicality – how and who.” *(local)* “Service configuration (i.e. who would provide and in which part of service)” *(local)*	
			Patients and parents play a role in PBM implementation		“[…] patients and parents being supportive as they could see the benefits” *(local)* “Facilitators: […] engagement from parents & CYP” *(local)*
			Different professional groups need to work together to implement PBM		“There has been discussion around this with the Oncology Dept. […]” *(local)* “Facilitators- […] Good working relationship with Oncology Service. […]” *(local)* “Facilitators: […] Department willing to try […] Support from [manufacturer]” *(local)* “Facilitators: Engagement with Haem onc [Haematology & Oncology] / BMTU [Bone Marrow Transplant Unit] teams […]” *(local)*
Optimism (The confidence that things will happen for the best or that desired goals will be attained)	Optimism Pessimism Unrealistic optimism Identity	-	Pessimism surrounding perception of provision of any treatment for mucositis management	“Barriers – […] therapeutic nihilism” *(individual, local) -*	
			Pessimism around engagement and acceptance from children and young people	“[…] Concerns about reluctance from patients (I don’t think these are founded on anything, but there is sometimes a feeling that adolescents won’t engage with therapies that require active participation” *(individual)*	
Beliefs about consequences (Acceptance of the truth, reality or validity about outcomes of a behaviour in a given situation)	Beliefs Outcome expectancies Characteristics of outcome expectancies Anticipated regret Consequents	Families and professionals need to feel there is a benefit	I do not believe PBM will have benefit for my patients	“proof of benefit […]” *(local, national)*	
			Where parents and children believe there is a benefit, this is helpful		“Facilitators: […] patients and parents being supportive as they could see the benefits” *(local)*
			I believe older children will be reluctant to engage with PBM	“[…] Concerns about reluctance from patients (I don’t think these are founded on anything, but there is sometimes a feeling that adolescents won’t engage with therapies that require active participation” *(national)*	
Intentions (A conscious decision to perform a behaviour or a resolve to act in a certain way)	Stability of intentions Stages of change model Transtheoretical model and stages of change	-	I am at the stage of pre-contemplation, without intention to implement	“Only heard about it as a possible thing – no plans to introduce and not sure how we’d go about it” *(local)* “Lack of awareness – unclear how would be implemented” *(individual, local, national)*	
			I am contemplating implementation but need support	“Would like to introduce but need to find dental team or others willing to be trained first! […]” *(local)* “Facilitators: Free kit to start with Department willing to try Support from THOR [manufacturer]” *(local)*	
Environmental context and resources (Any circumstance of a person’s situation or environment that discourages or encourages the development of skills and abilities, independence, social competence and adaptive behaviour)	Environmental stressors Resources/material resources Organisational culture/climate Salient events/critical incidents Person x environment interaction Barriers and facilitators	Resources required for implementation are diverse	There is no availability of a designated area for delivery of PBM	“Environmental to allocate designated areas for therapy” *(local)* “Barriers: […] suitable room (this was an initial barrier, but has been improved in our new hospital setting” *(local)* “ […] Facilities- i.e. lockable room” *(local)* “3 barriers – […], space, […]” *(local)* “Current building work which hopefully will be completed in Nov2021 [November 2021]” *(local)*	
			Availability of trained staff with time to deliver PBM is important	“[…] – Infrastructure required (equipment, training, staff)” *(local, national)* “Barriers: […]access to relevant training” *(local, national)* “Would like to introduce but need to find dental team or others willing to be trained first! […]” *(local)* “Barriers: sufficient numbers of trained staff”*(local)* “3 barriers – staffing, […]” *(local)* “Time Organisational” *(local)* “Time Relevant professionals […]” *(local)* “[…] Time to deliver […]” *(local)*	“Facilitators: […] Department willing to try […] *(local, national)* “Facilitators – enthusiastic technically competent staff […]” *(local)* “Strong dental links, manpower, […]” *(local)*
			Equipment needs to be available	“Barriers: […] reliable equipment” *(local, national)* “[…] – Infrastructure required (equipment, training, staff)” *(local, national)* “[…] Relevant professionals Equipment” *(local)*	“Facilitators: Free kit to start with […] Support from [ manufacturer]” *(local, national)* “Facilitators – […] access to approved ‘kit’” *(local)*
			Organisational support is important in implementation	“[…] Service configuration (i.e. who would provide and in which part of service)” *(local)* “Barriers: Medical devices approval Need SOP [Standard Operating Procedure] as not done before” *(local, national)* “[…] 2. Practicality – how and who. […]” *(local)* “Time Organisational” *(local)* “Barriers – […] NHS admin and therapeutic nihilism” *(local)*	“Facilitators: […] standardised protocols” (*local and national)*
			The cost of implementation will/will not be a barrier	“1. Cost […]” *(local)* “3 barriers – […] costs” *(local)* “Barriers: Funding /Capacity” *(local)* “[…] Cost of any equipment” *(local)* “Barriers – money, […]” *(local)*	“[…]Cost should not be prohibitive […]” (local) “Facilitators- […]Charitable funds” *(local)*
Social influences (Those interpersonal processes that can cause individuals to change their thoughts, feelings or behaviours)	Social pressure Social norms Group conformity Social comparisons Group norms Social support Power Intergroup conflict Alienation Group identity Modelling	The influence of different groups on each other is important	Working relationships between different stakeholders are important to implementation		“Refer to Glasgow if required” *(local)* “There has been discussion around this with the Oncology Dept. […]” *(local)* “Facilitators: Engagement with Haem onc [Haematology & Oncology] / BMTU [Bone Marrow Transplant Unit] teams engagement from parents & CYP” *(local)* “Facilitators- […] Good working relationship with Oncology Service. […]” *(local)*
			Departmental attitude towards PBM affects implementation	“Would like to introduce but need to find dental team or others willing to be trained first! […]” *(local)*	“Facilitators: […] Department willing to try […] *(local, national)* “Facilitators: very willing and keen dental colleagues, […]” *(local)* “Facilitators – enthusiastic […] staff […]” *(local)*
Emotion (A complex reaction pattern, involving experiential, behavioural, and physiological elements, by which the individual attempts to deal with a person significant matter or event)	Fear Anxiety Affect Stress Depression Positive/negative affect Burn-out	-	I have concerns around patient response	“[…] Concerns about reluctance from patients (I don’t think these are founded on anything, but there is sometimes a feeling that adolescents won’t engage with therapies that require active participation” *(national)*	

The most populated domains were knowledge, skills, social/professional role and identity, environmental context and resources and social influences. Overall, barriers were more frequently identified than facilitators.

No data could be coded to the domains of beliefs about capabilities, reinforcement, memory attention and decision processes or behavioural regulation. Two participants expressed an interest in exploring PBM further within a funded trial. However, from our data, the underlying motivation for trial involvement was not clear, and therefore these responses could not be accurately coded to the Theoretical Domains Framework.

#### Need for guidance to address lack of knowledge

A lack of awareness and knowledge around PBM for oral mucositis management was frequently reported as a barrier. Responders reported a lack of awareness of the use of PBM specifically for mucositis prevention. One responder referred to a lack of evidence-base, which was interpreted as a lack of awareness of the evidence-base and recommendations. Responders referred to a lack of guidance as a barrier and felt that production of standardised guidance would be a facilitator to implementation.

One responder reported support from colleagues with knowledge and previous experience of use of PBM was a facilitator.

#### Staff need access to training for skills acquisition

Responders felt that a lack of standardised training and protocols were a barrier to skills acquisition and, additionally, that a lack of wider training infrastructure and access to equipment presented a further barrier.

Where staff members had previous experience or were competent in delivering PBM, this was a facilitator coded to the skills domain.

#### Multiple, well identified groups need to work together towards implementation

Responders referred to different roles and teams including haematology, oncology, haematopoietic stem cell transplant and dental teams. Where these professional roles were working together, this was perceived as a facilitator to implementation. The role and identity of equipment manufacturers and children and young people and their parents was also referred to, where families were engaged this was viewed as a facilitator; support from equipment manufacturers was referred to, but it was unclear whether this was financial or in terms of training and skills development.

Many responders reported a lack of clearly defined professional roles and responsibilities in PBM as a perceived barrier to implementation. One responder referred to “relevant professionals” without an indication of who these professionals might be. Responders cited a barrier of the practicality of service configuration and which service would be responsible for implementation and delivery of PBM, whether this would be medical or dental teams.

#### Resources required for implementation are diverse

Environmental context and resources was the most frequently populated domain, with data predominantly pertaining to barriers. Some responders referred to specific resources, such as designated rooms for treatment, which are important for low-level laser systems. Wider resources identified included staffing, equipment, service funding, training and time. Where appropriate numbers of trained staff were present and funding could be provided, this was identified as a facilitator.

Organisational support was also seen as a valuable resource in terms of funding, staffing and protocol support for implementation of PBM service.

#### The influence of different groups on each other is important

Social influences between different professional roles involved in PBM delivery were highlighted, with good engagement between teams being identified as a facilitator. The influence of the dental team was coded to this domain; where a good relationship with the dental team was present, this was seen as a facilitator and where this was absent this was seen as a barrier.

Departmental attitude was seen as an important influence, with enthusiastic teams willing to implement a new service being seen as a key facilitator.

#### Families and professionals need to feel there is a benefit

Beliefs about consequences were a less frequently populated domain; however, this domain highlighted an important perceived barrier of acceptability to children and young people, particularly adolescents.

Where services were implemented, one responder reported that children and young people and their parents perceived a treatment benefit, which facilitated delivery of treatment.

### Levels of data

#### Individual

Data within the knowledge, social/professional role and identity and social influences domains was predominantly attributed to an individual responder level.

#### Local/organisational

Data within the skills and environmental context and resources domains were most frequently attributed to a local level, at the level of the hospital.

#### National/policy

Within the knowledge and skills domains, responders referred to the value of national guidance and standard operating procedures and training, which were attributed to a national level. Data relating to the professional role of a mentioned manufacturer within environmental resources and professional roles could also be ascribed to a national level.

## Discussion

This is the first published exploration of factors influencing PBM practices for mucositis management in CYP receiving cancer treatment in the UK. A 100% response rate was attained, which enables complete national analysis and identifies that this is a topic of interest to the principal treatment centres in the UK. Despite recommendation by multiple international bodies for the use of PBM as a supportive care therapy, this therapy is currently only available in two CCLG centres, both located in Scotland. However, a further four units indicated plans to implement a PBM service in the future.

Within the TDF analysis, data pertaining to knowledge, skills, social/professional roles and identity, environmental context and resources and social influences were most frequently identified. Barriers and facilitators within these domains were identified most frequently at a local level; however, many identified factors were attributed to a national level. To address identified barriers in knowledge and skills, a training programme could be developed to be implemented on a local or national level. The majority of units reported links with specialist Paediatric Dentistry services in relation to dental assessment. Liaison with these services was also perceived as a facilitator in implementing PBM services in the qualitative analysis. Paediatric Dentists are well positioned to support Paediatric Oncology teams in delivery of intra-oral therapies such as PBM. This research highlights the importance of such collaborations for both maintenance of oral health and mucositis management, and the value of involvement of the dental team as a facilitator of PBM implementation.

An unsupportive environmental context or lack of environmental resources was frequently identified as a barrier at an organisational level. Responders identified a lack of a designated room to deliver PBMn; with the advance of light emitting diode PBM, which does not require a designated laser room, it may be that this barrier is reduced. Initial start-up funding was identified as a barrier by many responders; it may be that this is related to the reported lack of knowledge and skills, with units being hesitant to fund a new service without appropriate personnel to train the relevant care teams. Impacts on staffing and time taken to deliver treatment were also highlighted as barriers within this domain, which will vary between individual units but is relevant in the wider context of the National Health Service.

Development of national collaborations between Paediatric Dentists and Paediatric Oncologists could support development of nationally standardised protocols and training resources. Such collaborations and resources could serve as a national facilitator to support CCLG principal treatment centres wishing to implement PBM therapy and may alleviate concerns regarding perceived barriers to implementation.

A lack of engagement or acceptability from children and young people, particularly adolescents, was reported as a perceived barrier at an individual patient level. Conversely, where children and young people had received treatment and could see benefit, their engagement was a facilitator to treatment provision. A small feasibility study of 13 patients receiving conditioning chemotherapy prior to haematopoietic stem cell transplant found good acceptability of extra-oral LED PBM [22]. However, this is a small sample consisting of adult and paediatric patients with a median age of 15 years. Further research is needed to explore the acceptability of PBM to children and young people of different age groups, with different treatment approaches and in different settings. Additionally, the acceptability to their parents and the healthcare professionals involved in treatment delivery should be explored. Wider approaches and resources such as provision of information videos or support groups may help to overcome concerns and advocate for this treatment.

Due to the survey nature of this research, barriers and facilitators to implementation were self-reported; responders may present a biased view and focus more on external rather than internal influences [23]. Qualitative interviews would have allowed a deeper exploration of the experiences of individual units in implementing PBM services. However, a cross-sectional survey allowed initial data capture on a national level. Responders may have exhibited a degree of response bias, given that they may have felt they were identifiable by the principal treatment centre they represent. However, it was necessary for responders to identify their CCLG unit to gain a geographical understanding of current PBM service provision.

## Conclusion

At the time of survey administration, PBM for mucositis management was only available to CYP in the UK receiving cancer treatment in Edinburgh and Glasgow. Several barriers and facilitators were identified which can be considered to primarily pertain to knowledge, skills, professional identify and role, social influence, and environmental context resources. Collaboration with paediatric dental teams was highlighted as a key facilitator in implementing PBM services. National networks could be utilised to overcome identified barriers at a national level and facilitate local PBM implementation.

## Data Availability

Data is available on request.

## Declarations

### Disclaimer

The views expressed in this publication are those of the author(s) and not necessarily those of the NIHR, the NHS or the Department of Health and Social Care.
